# Edible Film Preparation Using Chitosan/Gelatin/Phlorotannin-Embedded *Limosilactobacillus fermentum* FUA033 for Strawberry Preservation

**DOI:** 10.3390/foods15020381

**Published:** 2026-01-21

**Authors:** Jiaxuan Wang, Wenyue Ma, Yajian Su, Shu Liu, Ruyu Xu, Han Zhang, Xiaoyue Hou, Qiran Gu, Xu Zhao, Jiayi Hu, Yaowei Fang

**Affiliations:** 1 Jiangsu Key Laboratory of Marine Bioresources and Environment, Jiangsu Ocean University, Lianyungang 222005, China; 19825558738@163.com (J.W.); 19732894428@163.com (W.M.); syj661236@163.com (Y.S.); 2007000028@jou.edu.cn (S.L.); 15928116880@163.com (R.X.); 19519620486@163.com (H.Z.); 2020000057@jou.edu.cn (X.H.); 18961368820@163.com (Q.G.); 19518833559@163.com (X.Z.); 18012420539@163.com (J.H.); 2 Jiangsu Key Laboratory of Marine Biotechnology, Jiangsu Ocean University, Lianyungang 222005, China; 3 Co-Innovation Center of Jiangsu Marine Bio-Industry Technology, Jiangsu Ocean University, Lianyungang 222005, China; 4 School of Marine Food and Biological Engineering, Jiangsu Ocean University, Lianyungang 222005, China

**Keywords:** probiotic-encapsulated edible film, strawberry preservation, chitosan, gelatin, phlorotannins

## Abstract

In this study, we prepared edible films using chitosan/gelatin/phlorotannins (CGPs) embedded with probiotics and evaluated their preservation effects on strawberries. Edible films encapsulating *Limosilactobacillus fermentum* FUA033 (CGPFUA033) were prepared using the casting method. The intermolecular interactions, crystal structure, thermal stability, and morphology of the films, both prior to and following the incorporation of *L. fermentum* FUA033, were characterized using FT-IR, XRD, TG, and SEM analyses. The preservation efficacy of the edible films, with and without encapsulated *L. fermentum* FUA033, was assessed by monitoring the physical, chemical, and microbial properties, as well as the visual quality, of strawberries during a eight-day storage period. The results showed that encapsulation of *L. fermentum* FUA033 enhanced intermolecular interactions and thermal stability within the film matrix but did not significantly affect the crystalline structure of the edible film. At 0, 2, 4, 6, and 8 days, the CGPFUA033 treatment had preservation effects: the weight loss was 30.70 ± 1.53%, the total soluble solid content was 8.83 ± 0.28%, the decay index was 45.33 ± 1.53%, the malondialdehyde content was 7.44 ± 0.13 μmol/g, firmness was 21.49 ± 0.83 N, and the ascorbic acid content was 43.51 ± 0.79 mg/100 g. The shelf life of strawberries was extended by six days in the CGPFUA033 treatment group. Therefore, the chitosan/gelatin/phlorotannin edible film embedded with *L. fermentum* FUA033 has high preservation effects on strawberries, highlighting that *L. fermentum* FUA033 can be used as a probiotic for enhancing food preservation.

## 1. Introduction

Global fruit loss due to spoilage is as high as 10–15% [1]. Strawberries are categorized as highly perishable berries due to their high water content, delicate epidermis, and elevated respiratory intensity, making them particularly susceptible to mechanical damage, moisture loss, microbial infection, and nutritional degradation postharvest. In contrast, other fruits (such as apples and blueberries) possess denser epidermal structures and stronger storage tolerance, which limits the relative improvement of preservation effectiveness achievable through film-based technologies. Therefore, this study focuses on strawberries as a representative fruit model. To decrease waste, various fruit preservation technologies have been developed, including low-temperature storage, modified atmosphere packaging, irradiation, preservative additives, and edible films, all of which have high efficacy [2]. Among them, edible films composed of natural biopolymers are formed through intermolecular interactions inherent to the biopolymers themselves, including electrostatic interactions, hydrogen bonding, hydrophobic interactions, among others. These films inhibit microbial growth and regulate the exchange of oxygen, carbon dioxide, and moisture, making them widely applicable in fruit preservation. As they are biodegradable and have inherent antimicrobial properties, researchers have considered them for application in postharvest technology [3].

Edible films are classified primarily by their matrix origin into polysaccharides, proteins, and lipids [4]. Polysaccharides include primarily chitosan, cellulose, and starch. Among these, chitosan, owing to its antimicrobial properties, biocompatibility, and stability, is considered to be the optimal carrier for edible films [5]. Proteins primarily include gelatin, collagen, and whey protein, among which gelatin serves as a versatile raw material for the preparation of edible films because of its excellent film-forming properties [6]. Lipids primarily consist of waxes, fatty acids, and oils. Among these materials, waxes have excellent hydrophobicity, effectively preventing water permeation [7]. During storage, fruits undergo significant volumetric and textural changes, making them highly susceptible to microbial contamination due to physical damage. Therefore, incorporating phenolic extracts, plant extracts, or essential oils into edible films can considerably increase their antimicrobial activity [8]. However, the antioxidant and antimicrobial properties of the edible films currently available for fruit preservation need to be improved [9].

Probiotics metabolically produce bioactive compounds such as organic acids, bacteriocins, and exopolysaccharides. The incorporation of probiotics into edible films enhances antimicrobial and antioxidant activities, decreases moisture and nutrient loss, and ultimately improves fruit preservation efficacy [10]. Moreover, probiotics have multifaceted bioactive properties that not only extend the shelf life but also increase the nutritional value of fruits [11]. *Limosilactobacillus fermentum* FUA033, previously screened by our research group, can convert ellagic acid in fruits into urolithin A [12], which is a metabolite with antioxidant [13], gut function-modulating, immune-regulatory, musculoskeletal health-promoting, and metabolic health-enhancing activities [14]. This strain is extremely safe and has desirable probiotic properties [15]. However, the viability of probiotics in edible films is strongly influenced by the matrix composition and film-forming methods, with limited research available on this key aspect [16].

Although research on probiotic-based edible films has made progress, limitations still exist. Firstly, existing studies have mostly focused on drying-based film formation, which can severely damage probiotic viability. Secondly, current films lack additional bioactive functions. To address these scientific gaps, this study employs room-temperature film preparation to provide a theoretical basis for low-temperature processing. Additionally, by combining probiotics with the bioactive substance phlorotannin, an active film with both probiotic and bioactive properties was developed, expanding the application scope of such films. The edible film containing *Lactobacillus mucilaginosus* FUA033 was prepared in this study. The structure of the edible film was characterized by using chitosan, gelatin and brown algae polyphenol as the base material. The storage experiment of strawberry was carried out to determine the effect of the edible film on the appearance, physical and chemical properties and microbial characteristics of strawberry during storage.

## 2. Materials and Methods

### 2.1. Materials and Chemicals

*Limosilactobacillus fermentum* FUA033 was screened and preserved in our laboratory and deposited at the China General Microbiological Culture Collection Center (CGMCC No. 28447). Chitosan (deacetylation degree ≥ 95%, viscosity: 50–800 MPa·s, Sinopharm Chemical Reagent Co., Ltd., Shanghai, China), gelatin (Sinopharm Chemical Reagent Co., Ltd., Shanghai, China), phlorotannins (purity ≥ 98%, Sinopharm Chemical Reagent Co., Ltd., Shanghai, China), glacial acetic acid (Sinopharm Chemical Reagent Co., Ltd., Shanghai, China), glycerol (Macklin Biochemical Technology Co., Ltd., Shanghai, China), and strawberries were purchased from a local market (Julong North Road, Lianyungang, Jiangsu, China) and were selected for freshness, absence of mechanical damage, and uniform size.

### 2.2. Preparation of the Bacteria

The cryopreserved *L. fermentum* FUA033 was retrieved from a freezer at –80 °C, thawed at room temperature for 3 min, and transferred to a biosafety cabinet. The strain was activated on MRS solid medium and cultured at 37 °C for 24 h. A single colony was inoculated in MRS liquid medium and incubated in an anaerobic jar at 37 °C for 24 h. The bacterial suspension was washed three times with sterile saline at 12,000× *g* for 3 min each to remove residual medium, and the pelleted cells were collected for subsequent use.

### 2.3. Preparation of Edible Films

The edible films were prepared using the solvent casting method [17]. Brown algae polyphenol (0.6 g) was first dissolved in 100 mL of 2% (*v*/*v*) acetic acid, followed by the addition of chitosan (2 g) and glycerol (1 g) under continuous stirring for 1.5 h. Subsequently, gelatin (2 g) was incorporated, and stirring was continued for an additional 2 h. The resulting solution was subjected to ultrasonic degassing, after which 24 g of the mixture was poured into a 12 cm × 12 cm mold and dried at room temperature to form an edible film, designated as CGP. The centrifuged cell precipitate of *Lactobacillus fermentum* FUA033, obtained post-cultivation, was added to the film-forming solution at a concentration of 1.5% (*w*/*v*) and thoroughly mixed. The mixture was then processed again via ultrasonic degassing, and the resulting composite material was designated as CGPFUA033. Chitosan (deacetylation degree ≥ 95%) exhibits better solubility and film-forming properties, with a concentration of 2% *(w*/*v*) being the commonly effective film-forming concentration [18]. The ratio of gelatin to chitosan at 1:1 can produce excellent mechanical properties and a uniform microstructure [19]. The final addition amount of brown algae polyphenols and microbial biomass was determined through preliminary experiments.

### 2.4. Characterization of Edible Films

The edible films were characterized following the methodology [20]:

**Fourier transform infrared (FT-IR) spectroscopy**

Fourier transform infrared (FT-IR) spectra were acquired using a Nicolet-iS10 spectrometer (Thermo Fisher Scientific, Shanghai, China) and potassium bromide tablets, with scanning performed over the range of 4000–400 cm^−1^ at a resolution of 4 cm^−1^, 32 scans.

**X-ray diffraction (XRD) analysis**

X-ray diffraction (XRD) analysis was performed using a Bruker D8 Advance diffractometer (Bruker Corporation, Karlsruhe, Germany) operated at 40 kV and 40 mA, with scanning conducted over the 2θ range of 5–60° at a rate of 2°/min.

**Thermogravimetric (TG) analysis**

Thermogravimetric (TG) analysis was conducted using an STA 449 F3 simultaneous thermal analyzer (NETZSCH, Bavaria, Germany). The samples (5–10 mg) were heated from 30 °C to 800 °C at a rate of 20 °C/min under a nitrogen atmosphere.

**Morphological observation (scanning electron microscopy, SEM)**

Morphological features were observed using a JSM-6390LA scanning electron microscope (SEM) (Carl Zeiss, Oberkohen, Germany) at an accelerating voltage of 5 kV to characterize the surface microstructure of the edible films. Five randomly selected regions of each film sample were observed by SEM, with the images shown in this paper being the most representative results.

### 2.5. Research on the Use of Edible Films for Strawberry Preservation

Strawberries were rinsed with sterile water and divided into three groups. The fruits were immersed for 3 min in sterile water, chitosan/gelatin/phlorotannins coating solution, or chitosan/gelatin/phlorotannins/*L. fermentum* FUA033 coating solution, followed by air-drying in a biosafety cabinet. The samples were stored in disposable polypropylene containers and maintained in an incubator at 25 °C [21] with 80 ± 2% relative humidity (Since harvesting is conducted in environments with high temperature and humidity, strawberries are more prone to spoilage under elevated temperatures). Measurements were conducted at 0, 2, 4, 6, and 8 days [22].

#### 2.5.1. Physical Measurements of Strawberries

Weight loss

The weight loss of strawberries was determined by calculating the difference between the initial weight and daily weight measurements using the following formula [23]:

$$Weight\;loss\left(\%\right)=\frac{m_{0}-m_{1}}{m_{0}}\times 100\%$$

Here, *m*_0_ is the pre-storage mass, and m_1_ is the post-storage mass.

Hardness

Firmness was measured using a TMS-Pro texture analyzer (Yingsheng Technology Co., Ltd., Shanghai, China). The test parameters included a cylindrical probe (P5); pre-test, test, and post-test speeds of 2, 1, and 2 mm/s, respectively; and a 50% compression depth [24].

#### 2.5.2. Chemical Measurements of the Strawberries

pH

The strawberries were homogenized into juice and filtered through gauze. The pH was measured using a PHS-3C pH meter (Shanghai Yidian Physical Optical Instrument Co., Ltd., Shanghai, China).

Titratable acidity (TA)

The titratable acidity was determined by the acid–base titration method [25], and the strawberries were crushed and homogenized with 50 mL of distilled water. The filtrate was obtained through filtration, and two drops of 1% (*w*/*v*) phenolphthalein indicator were added to 10 mL of the filtrate. Titration was performed using 0.1 mol/L NaOH until a permanent pink endpoint was achieved. The formula used to calculate TA is as follows:

$$\mathrm{TA}\;\mathrm{content}\left(\%\right)\;=\;\frac{V\times V_{1}\times C\times K_{c}}{V_{2}\times m}\times 100$$

Here, *C* represents the molar concentration of NaOH, while *V*_1_, *K*, *m*, *V*_2_, and *V* represent the volume of NaOH consumed, the conversion factor (0.064 for citric acid equivalent), the mass of the strawberry sample, the volume of the aliquot used for titration, and the total volume of the diluted sample, respectively.

Total soluble solids (TSS)

The strawberry juice was dropped onto the prism of a WZS handheld refractometer (Shanghai Yidian Physical Optical Instrument Co., Ltd., Shanghai, China), and readings were recorded [26].

Ascorbic acid content

First, 10 g of strawberry was homogenized with 10 mL of oxalic acid solution (5 g/L) in a mortar. The mixture was diluted to 100 mL with distilled water and filtered [27]. A 2.0 mL aliquot of the extract was transferred to a quartz cuvette, and the absorbance was recorded at 242 nm using oxalic acid solution as the blank. The results were expressed as mg/100 g:

$$Ascorbic\;acid\;content\left(\mathrm{mg}/100g\right)\;=\;\frac{V\;\times \;m}{V_{s}\times W\times 1000}\times 100$$

Here, *m*, *V*, *VS*, and *W* represent the mass of ascorbic acid, total volume of sample extract, volume of extract used for the assay, and sample mass, respectively.

Malondialdehyde (MDA)

Following the method described by Jin et al. [28], 2.0 g of strawberry was homogenized with 4 mL of trichloroacetic acid (100 g/L), and the supernatant was collected after centrifugation at 10,000× *g* for 12 min at 4 °C. The supernatant was mixed with thiobarbituric acid solution at a 1:1 (*v*/*v*) ratio. The TCA mixture was used as the blank, and the mixture was vortexed, boiled for 15 min, cooled, and centrifuged. The absorbance was measured at 450 nm, 532 nm, and 600 nm.

Peroxidase (POD) activity

First, 1 g of the strawberry sample was homogenized with 4 mL of extraction buffer in an ice bath, followed by centrifugation at 12,000× *g* for 10 min at 4 °C. The supernatant was collected, and POD activity was assayed using the POD detection kit (Aidisheng, Yancheng, China), following the manufacturer’s instructions.

#### 2.5.3. Strawberry Decay Index and Microbial Analysis

Decay index

The decay index (DI) was evaluated using disease severity scoring [25]. The extent of visible decay on fruit surfaces was graded as follows: 0 = no decay, 1 = 1–10% decay, 2 = 11–25% decay, 3 = 26–40% decay, 4 = 41–50% decay, and 5 = >50% decay.

$$\mathrm{Decay}\;\mathrm{index}\;(\%)=\frac{\sum \;(\mathrm{Severity}\;\mathrm{grade}\;\times \;\mathrm{number}\;\mathrm{of}\;\mathrm{fruits}\;\mathrm{in}\;\mathrm{the}\;\mathrm{corresponding}\;\mathrm{grade})}{\left(\mathrm{highest}\;\mathrm{grade}\;\times \;\mathrm{total}\;\mathrm{number}\;\mathrm{of}\;\mathrm{fruits}\right)}\times 100\%$$

Microbial analysis

Microbial analysis was performed following a previously described method with modifications [29]. Next, 5 g of the strawberry sample was homogenized with 45 mL of sterile saline (0.9% *w*/*v*) for 10 min. The homogenate was serially diluted (10^−1^ to 10^–6^), and 1 mL aliquots of each dilution were pour-plated onto plate count agar (PCA). After incubation at 37 °C for 48 h, colonies were counted and expressed as log CFU/g.

### 2.6. Statistical Analysis

The experimental unit of this study was individual strawberries, with all measurements based on independent strawberry samples. A completely randomized design was adopted, with strawberry types categorized as CK, CGP, and CGPFUA033, and storage times set at 0, 2, 4, 6, and 8 days. Time was not treated as a repeated measure, and the data were expressed as the mean ± standard deviation. The differences among and between groups were determined by conducting one-way analysis of variance (ANOVA) in IBM SPSS Statistics 25 (Chicago, IL, USA). All differences were considered to be statistically significant at *p* < 0.05. Data processing and chart drawing were carried out using Origin 2021 software.

## 3. Results and Discussion

### 3.1. Characterization of Edible Films

**Figure 1:**
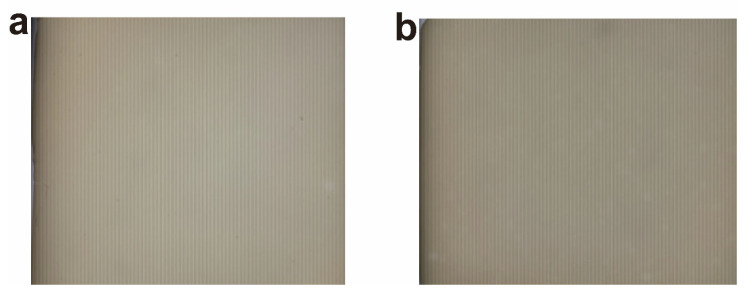
Schematic diagrams of edible films: (**a**) CGP and (**b**) CGPFUA033.

**Figure 2:**
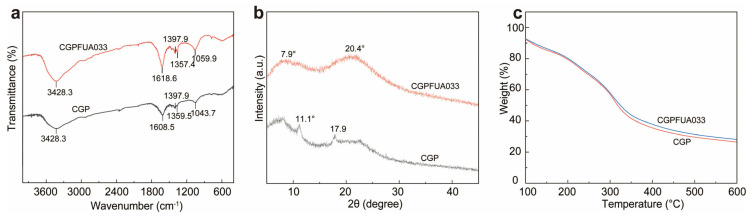
Characterization of CGP and CGPFUA033 edible films: (**a**) FTIR spectra, (**b**) XRD patterns, and (**c**) TGA thermograms.

We performed FTIR spectroscopy to track the functional groups and intermolecular interactions within the edible films (Figure 1 and Figure 2a). The characteristic absorption band at 3500–3300 cm^−1^ was attributed to overlapping O-H stretching vibrations and N-H symmetric stretching [24]. Both films exhibited O-H stretching at 3428.3 cm^−1^, indicating the presence of intermolecular interactions. The identical peak positions of CGPFUA033 and CGP indicated that incorporating *L. fermentum* FUA033 did not significantly alter the hydrogen-bonding network, confirming successful film preparation. Characteristic peaks at 1608.5 cm^−1^ (for CGP) and 1618.6 cm^−1^ (for CGPFUA033) indicate the stretching vibration of the amide I band (C=O). Weak peaks appearing at 1357.4 cm^−1^ (for CGPFUA033) and 1359.5 cm^−1^ (for CGP) correspond to the stretching vibration of the amide III band (C-N). Additionally, peaks at 1043.7 cm^−1^ (for CGP) and 1059.9 cm^−1^ (for CGPFUA033) demonstrate C-O-C stretching. These observations suggest that the incorporation of *L. fermentum* FUA033 led to interactions within the edible film, thereby altering its microenvironment. No significant new characteristic peaks were detected after the addition of probiotics, indicating that *Lactobacillus fermentum* FUA033 did not significantly disrupt the existing molecular networks or intermolecular interactions in the edible film.

XRD patterns were used to investigate the crystalline structure of the edible films and to evaluate their compatibility (Figure 2b). The CGP film exhibited a distinct peak at 2θ = 11.1°, indicating the presence of a certain degree of ordered crystalline structure. The peak observed at 2θ = 17.9° may correspond to gelatin overlapping with the weak crystalline signal of chitosan. After the incorporation of *L. fermentum* FUA033, the XRD pattern of the CGPFUA033 film showed that the peak around 2θ = 20° exhibited reduced intensity and increased full width at half maximum. This suggests a decrease in crystalline ordering, which typically implies that the addition of probiotics may have disrupted the molecular chain arrangement within the chitosan-gelatin network, leading to reduced ordering in the crystalline regions [30]. However, no distinct new diffraction peaks were observed, indicating that FUA033 did not introduce new crystalline phases or cause significant phase separation. Combined with the comprehensive analysis of FTIR and SEM results, we propose that the interaction between FUA033 and the film matrix is primarily physical in nature. This suggests good macroscopic compatibility between the two components, rather than a chemical-bonding-induced reorganization of the crystalline structure.

The TGA thermograms of CGP and CGPFUA033 are illustrated in Figure 2c. Both films underwent three primary thermal degradation stages: (1) 100–150 °C: The evaporation of adsorbed and free water contributed to the initial mass loss. Owing to its enhanced water absorption capacity, CGPFUA033 exhibited a slightly greater initial mass loss compared to CGP [31]. (2) 150–300 °C: The higher mass loss rate was attributed to the volatilization of glycerol, oxidative degradation of phlorotannins, and scission of small molecules. The slower mass loss rate of CGPFUA033 indicated a delayed volatilization of small molecules and a retarded degradation of phlorotannins in the edible film. (3) 300–400 °C: The cleavage of chitosan and gelatin backbones, along with the weakening of hydrogen bonds between chitosan and gelatin, promoted the pyrolysis of phlorotannins, thereby further increasing the mass loss [32]. The delayed decomposition peak of CGPFUA033 indicated that the incorporation of *L. fermentum* FUA033 could enhance thermal stability. (4) 400–600 °C: The thermal stability of CGPFUA033 was 1.56% higher than that of CGP, which was attributed to the carbonization of phlorotannins and the decomposition of residues. The inorganic components of *L. fermentum* FUA033, combined with the aromatic structure of phlorotannins, facilitated the formation of a stable char layer at high temperatures, thereby significantly enhancing the thermal stability of CGPFUA033.

**Figure 3:**
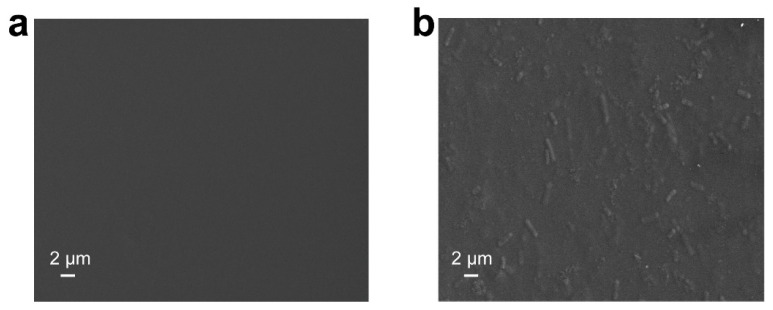
Scanning electron microscopy (SEM) images of edible film surfaces: (**a**) CGP and (**b**) CGPFUA033.

The CGP and CGPFUA033 solutions were cast into molds using the solvent casting method to prepare edible films. The SEM images (Figure 3a) revealed the homogeneous and compact microstructures of the films, indicating excellent molecular compatibility among the components [33]. In contrast, the incorporation of *L. fermentum* FUA033 increased surface roughness (Figure 3b). The probiotic cells were uniformly dispersed as rod-shaped particles (in the µm range) within the film matrix without visible aggregation or phase separation. This finding indicated that the compatibility between *L. fermentum* FUA033 and the chitosan/gelatin/phlorotannins film-forming matrix was high.

### 3.2. Physical Properties of Strawberries

**Figure 4:**
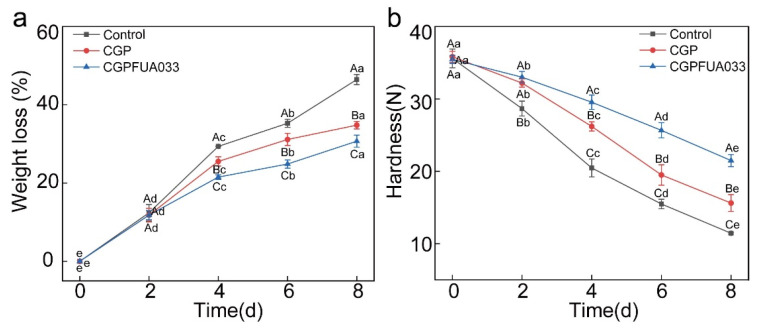
Effects of different treatments on physical properties during strawberry storage: (**a**) weight loss rate and (**b**) hardness. Note: In the figure, the small letters represent comparisons within the same treatment group at different storage times, whereas the capital letters represent comparisons between different treatment groups at the same storage time. Different letters indicate significant differences (*p* < 0.05). The same applies to this text.

During storage, the weight loss of strawberries is attributed primarily to the evaporation of moisture through epidermal stomata [8]. In this study, the initial weight loss was 0% for all groups (Figure 4a), with a progressive increase in weight loss due to respiration and evaporation of water. Weight loss in the control group increased considerably after day 2, reaching 46.46 ± 1.29% at the end of storage, as direct environmental exposure accelerated moisture loss [34]. In contrast, the CGP and CGPFUA033 groups exhibited slower weight loss after day 4, with CGPFUA033 achieving the lowest final weight loss rate of 30.70 ± 1.53%. This suggests that the combined use of *L. fermentum* FUA033 with chitosan/gelatin/phlorotannin coatings contributed to a significant reduction in weight loss (*p* < 0.05). The observed effect may be associated with the potential antimicrobial synergy between chitosan and metabolites of *L. fermentum* FUA033, as well as the complementary preservative function of phlorotannins. Together, these components likely acted to inhibit microbial growth, thereby reducing decay-related juice leakage and associated nutrient loss. Rahmati-Joneidabad et al. similarly reported enhanced weight retention in strawberries treated with *Limosilactobacillus reuteri* and cress seed extract [11].

Firmness serves as a critical indicator of fruit storability and shelf life [35]. The firmness of all strawberry samples decreased (Figure 4b), which occurred due to an acceleration of tissue maceration caused primarily by the microbial secretion of pectinases or cellulases and the activation of endogenous enzymes. The firmness of the samples in the control group was significantly lower than that of the samples in the CGP and CGPFUA033 groups, which indicated that the most severe softening occurred due to the absence of protective coatings. In contrast, the samples in the CGP and CGPFUA033 groups presented the highest firmness values of 15.61 ± 0.16 N and 21.49 ± 0.83 N, respectively, at the end of storage. The observed preservation effect may be attributed to the coating’s ability to inhibit microbial proliferation by limiting the availability of substances required for microbial growth. This likely contributed to the reduction in infection-induced softening and respiratory metabolism. Furthermore, CGPFUA033 significantly inhibited the decline in firmness (*p* < 0.05), which is consistent with the delayed softening observed in strawberries [36]. Wu et al. also reported an increase in firmness in chitosan-sweet potato anthocyanin-coated strawberries [37]. Although the water barrier properties of the coating were not directly measured in this study, the significantly reduced weight loss and better-maintained firmness of strawberries coated with CGPFUA033 indicate that the CGPFUA033 coating functioned as an effective physical barrier.

### 3.3. Chemical Properties of Strawberries

**Figure 5:**
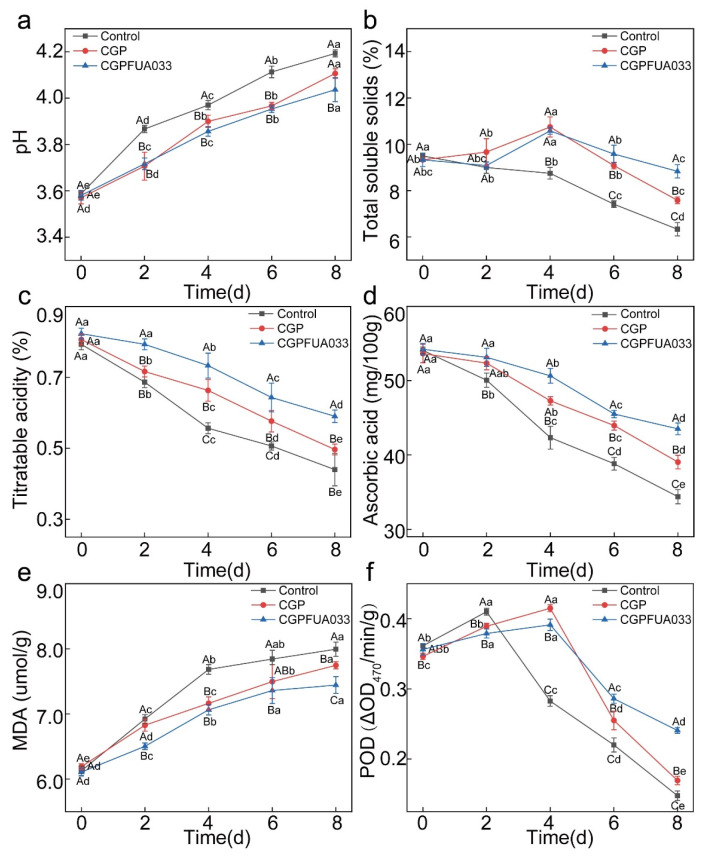
Effects of different treatments on chemical properties during strawberry storage: (**a**) pH, (**b**) total soluble solids, (**c**) titratable acidity, (**d**) ascorbic acid content, (**e**) malondialdehyde content, and (**f**) peroxidase activity.

The pH of fruits serves as a direct indicator of fruit ripening progression. During maturation, the gradual decrease in organic acids through respiratory metabolism and their conversion to sugars leads to a natural decline in acidity. The strawberry samples showed an initial pH of 3.58 ± 0.02, which progressively increased during storage (Figure 5a). This increase in pH resulted from combined factors, including organic acid degradation and alkaline metabolites produced by microbial activity. Among all treatment groups, the uncoated control showed the greatest increase in pH, reaching 4.19 ± 0.01 by the end of storage. The CGP group maintained intermediate values at 4.10 ± 0.02, whereas the CGPFUA033 group showed greater pH stability, with a final value of 4.03 ± 0.05. The CGPFUA033 treatment resulted in significantly lower pH values compared to both the control and CGP treatments (*p* < 0.05). This decrease in pH may be associated with the combined antioxidant capacity of chitosan and phlorotannins, which could help reduce oxidative degradation of acidic components and slow the consumption of organic acids. Additionally, the presence of *L. fermentum* FUA033 and its metabolic activity may have contributed to a lower pH microenvironment through the gradual release of acidic substances, collectively enhancing the preservative function of the coating [38]. This combined mechanism effectively delayed senescence and decay, thereby extending the shelf life of strawberries.

The TSS content represents one of the primary quality indicators for fruits and is strongly correlated with sugar and organic acid concentrations. The control group showed a time-dependent decrease in the TSS content during storage (Figure 5b). This reduction primarily resulted from the increase in metabolic and respiratory activities in the strawberries in the control group exposed to high-oxygen conditions, leading to accelerated consumption of sugars as respiratory substrates. This gradual decrease in the TSS content during extended storage represents a characteristic physiological response in strawberry fruits [39]. The CGP and CGPFUA033 groups presented a characteristic pattern of initial increase followed by a decrease in the TSS content, peaking on day 4 of storage, with values of 10.58 ± 0.14% and 10.75 ± 0.43%, respectively, before showing a significant reduction. This biphasic trend likely reflects the dynamic metabolic shifts during storage, where the initial starch-to-sugar conversion was subsequently followed by respiratory consumption of soluble sugars. This observation aligns with the former findings [40]. The CGPFUA033 group showed the highest TSS content (8.83 ± 0.28%) at the end of the storage period, demonstrating a significant reduction in the rate of decrease in TSS (*p* < 0.05) and better preservation of organic acids compared to the other treatment groups. The enhanced preservation effect observed in the CGPFUA033 group may be explained by the integrated action of several contributing factors. The chitosan-gelatin matrix formed a semi-barrier coating that reduced oxygen permeability, thereby limiting aerobic respiration in the fruit tissues. Meanwhile, the inclusion of phlorotannins helped alleviate oxidative stress, which could influence carbohydrate dynamics. In addition, the presence of *L. fermentum* FUA033 appeared to restrict mold development, potentially reducing sugar depletion linked to localized decay. Together, these factors likely contributed to the overall quality retention observed in strawberries treated with CGPFUA033.

Strawberries contain organic acids such as citric acid and malic acid, which contribute to the acidity and flavor of the fruit [41]. The metabolic rate of strawberries significantly influences changes in TA levels. During storage, strawberry senescence leads to the respiratory consumption of organic acids, resulting in a decrease in acidity. The TA of all strawberry samples decreased (Figure 5c), as organic acids were consumed during respiration. The control group, which lacked a protective coating, showed the greatest decrease in TA (from 0.79 ± 0.01% to 0.44 ± 0.04%); this was attributed to accelerated metabolism under high-temperature conditions, where organic acids are extensively broken down as substrates in the TCA cycle to supply energy. At the end of storage, the TA values of the CGP group and CGPFUA033 group were 0.49 ± 0.01% and 0.59 ± 0.02%, respectively. The CGPFUA033 group showed the highest titratable acidity (TA) and the slowest decline in acidity. This trend may be associated with several factors. The coating formed by CGPFUA033 likely limited oxygen availability, which could reduce aerobic respiration and slow the metabolic utilization of organic acids. Furthermore, the antimicrobial activities of chitosan and phlorotannins may have contributed to inhibiting fungal growth, thereby delaying fruit senescence. The presence of *L. fermentum* FUA033 might also help restrict the activity of acid-degrading microorganisms, potentially reducing exogenous enzymatic breakdown of acids. These findings are largely consistent with the arguments proposed by Sani et al. In a study, chitosan and zinc oxide nanoparticles were used to extend the shelf life of strawberries [42].

The ascorbic acid content serves as a key indicator for evaluating the antioxidant capacity and nutritional value of strawberries [43]. The ascorbic acid content decreased significantly during storage (Figure 5d), as it underwent oxidative degradation in strawberries. The fruit contains ascorbic acid oxidase and polyphenol oxidase, which come into contact with ascorbic acid and catalyze its oxidation following cellular damage. The ascorbic acid content in the control group substantially decreased (from 54.07 ± 0.45 mg/100 g to 34.38 ± 0.94 mg/100 g). This pronounced decrease occurred because the strawberries in the control group were directly exposed to the environment, making them susceptible to oxidation reactions and enzymatic degradation through contact with oxygen and microorganisms. During storage, the CGP and CGPFUA033 groups maintained significantly greater ascorbic acid contents than the control group (*p* < 0.05), effectively mitigating the degradation of ascorbic acid. The phlorotannins present in the coating were found to suppress the activity of ascorbic acid oxidase. The CGPFUA033 group presented the greatest retention of ascorbic acid throughout storage, with the most gradual decline rate, which is consistent with conclusions previously reported [44]. This result indicates that the coating formulation containing *L. fermentum* FUA033 was the most effective in preserving ascorbic acid levels in strawberry fruits during storage.

Malondialdehyde, a key byproduct of membrane lipid peroxidation in plants, is generated through the oxidative degradation of unsaturated fatty acids in cell membranes under reactive oxygen species attack. The MDA content in all strawberry groups increased during storage (Figure 5e), indicating postharvest disruption of cell membrane integrity. This damage to the membrane promotes ROS accumulation, exacerbating lipid peroxidation and consequently leading to progressive accumulation of MDA. The control group presented the highest MDA level (7.99 ± 0.11 μmol/g), indicating the most severe impairment of the membrane system, where the cellular structure and physiological functions were significantly compromised. These results confirmed that sterile water treatment fails to inhibit the rapid deterioration of quality in stored strawberries. The CGP group presented an MDA content of 7.74 ± 0.05 μmol/g on day 8, which was significantly lower than that of the control group, although it still demonstrated an increase in MDA levels. This protective effect can be attributed to the physical barrier formed by CGP, which reduces exposure to oxygen and loss of moisture, thereby suppressing MDA generation. Additionally, the chitosan and phlorotannins in CGP showed antioxidant properties by scavenging free radicals and inhibiting lipid peroxidation in strawberries. At the end of the storage period, the CGPFUA033 group maintained the lowest MDA level at 7.44 ± 0.13 μmol/g, which was significantly lower than that of both the control and CGP groups, with the most gradual increase rate (*p* < 0.05). These findings align with those reported by Yu et al. [45]. The results indicated that the incorporation of *L. fermentum* FUA033 effectively suppressed the accumulation of MDA in strawberry fruits. This reduction in MDA, a marker of membrane lipid peroxidation, was correlated with a measured decrease in ROS levels. These combined observations suggest that treatment with *L. fermentum* FUA033 was associated with diminished oxidative stress and delayed fruit senescence.

Peroxidase is a crucial enzyme in the fruit antioxidant system that catalyzes the decomposition of H_2_O_2_ into H_2_O, thereby eliminating reactive oxygen species and protecting cells from oxidative damage [46]. During storage, all strawberry samples showed a characteristic pattern of initial increase followed by a gradual decrease in POD activity (Figure 5f). In the control group, POD activity peaked on day 2 at 0.41 ΔOD_470_/min/g, after which it decreased steadily from day 4. This trend suggests that oxidative stress in untreated strawberry plants initially activated POD production, leading to a rapid increase in POD activity, followed by accelerated oxidative processes that ultimately decreased POD activity. Compared to the control group, the CGP and CGPFUA033 groups presented a significantly slower decline in POD activity after day 4 but maintained relatively greater POD activity. This phenomenon may be attributed to the following mechanisms. The chitosan/gelatin coating formed a physical barrier that decreased oxygen permeability, thereby decreasing reactive oxygen species generation and subsequent activation of POD. The antioxidant properties of phlorotannins enable them to directly scavenge ROS and decrease the accumulation of H_2_O_2_, which serves as the substrate for POD activity. The POD activity of the CGPFUA033 group increased before day 4, and the highest POD activity was 0.241 ± 0.004 ΔOD_470_/min/g at the end of storage. The results indicated that the CGPFUA033 treatment catalyzed the decomposition of H_2_O_2_ in strawberry tissues more effectively, thereby reducing its excessive accumulation and associated peroxidative damage. This protective effect coincided with a measured reduction in POD activity. The combined reduction in H_2_O_2_ levels and POD activity suggests an effective mitigation of oxidative stress, which may be further supported by the treatment’s documented antimicrobial properties. These findings were consistent with the results reported by Gautam et al., who investigated quality preservation in strawberries using xanthan gum-sodium nitroprusside composite coatings [47].

### 3.4. Decay Index and Microbial Properties of Strawberries

**Figure 6:**
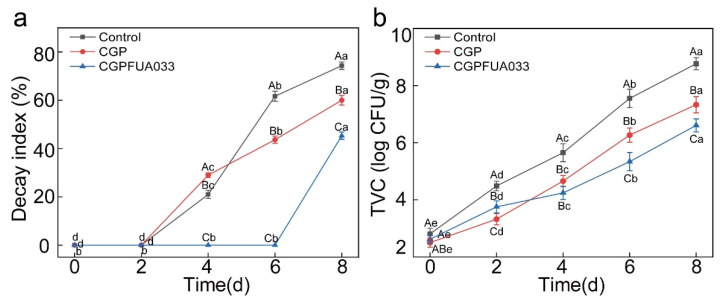
Effects of different treatments on the (**a**) decay index and (**b**) total viable count (TVC) during the storage of strawberries.

Strawberries easily soften during storage due to physiological changes and microbial infections, which significantly shorten their shelf life [48]. The decay indices of all strawberry samples increased throughout storage (Figure 6a). By the end of the storage period, the control group showed the highest DI (74.33 ± 1.53%), demonstrating the most dramatic deterioration. This accelerated decay occurred because unprotected strawberries were exposed to the environment, which allowed microorganisms to rapidly proliferate through surface colonization or mechanical wounds, leading to characteristic spoilage symptoms, including mold spots, tissue softening, and water leakage. Compared to the control group, the CGP and CGPFUA033 groups showed significantly lower decay indices of 60.00 ± 2.00% and 45.33 ± 1.53%, respectively. This protective effect may be attributed to the ability of the edible coating to physically block oxygen penetration and microbial contact with strawberry surfaces; the cationic nature of chitosan disrupted microbial cell membranes, thereby inhibiting the proliferation of pathogens. The phenolic hydroxyl groups in phlorotannins effectively suppressed pathogenic enzyme activity and scavenged free radicals, collectively delaying oxidative membrane damage-induced decay. The CGPFUA033 group showed a slower increase in the decay index (DI) during late storage. This corresponded with a significantly lower decay index in strawberries treated with the chitosan-based coating enriched with *L. fermentum* FUA033 compared to the control (*p* < 0.05), indicating its effectiveness in suppressing fungal-induced decay [29]. These findings align with the results reported, in which coatings derived from *H. illucens* similarly demonstrated effective fungal decay suppression and extension of shelf life in fresh produce [49].

The total viable count serves as a critical indicator for evaluating microbial contamination in fruits, reflecting their overall quality and safety. The TVC of all groups progressively increased due to microbial proliferation during storage (Figure 6b). The initial TVC of strawberries ranged from 2.50 to 2.80 log CFU/g, which is consistent with the 2.5–2.75 log CFU/g [50]. The control group showed the most dramatic microbial growth, reaching 8.76 ± 0.21 log CFU/g at the storage endpoint, substantially exceeding the international safety threshold of 5–6 log CFU/g [51]. Compared to the control group, the CGP and CGPFUA033 groups presented slower TVC growth rates. The CGPFUA033 group presented higher TVC values than the CGP group on day 2, probably because the presence of *L. fermentum* FUA033 contributed to the initial microbial count. However, after four days of storage, the CGPFUA033 group presented significantly lower TVC levels than the control and CGP groups (*p* < 0.05). These results indicated that CGPFUA033 effectively suppressed the growth and proliferation of total viable bacteria in strawberries. These findings may be attributed to multiple synergistic mechanisms: the chitosan-gelatin composite formed a protective barrier that physically prevents microbial invasion; the cationic nature of chitosan electrostatically disrupted the integrity of the bacterial membrane; chitosan and *L. fermentum* FUA033 showed complementary antimicrobial effects; and phlorotannins reduced microbial metabolic byproducts through their antioxidant effects. CGPFUA033 significantly prolonged the shelf life of strawberries (*p* < 0.05), which aligns with the results reported by Barzegar et al. [52].

### 3.5. Visual Quality Evaluation of Preserved Strawberries

**Figure 7:**
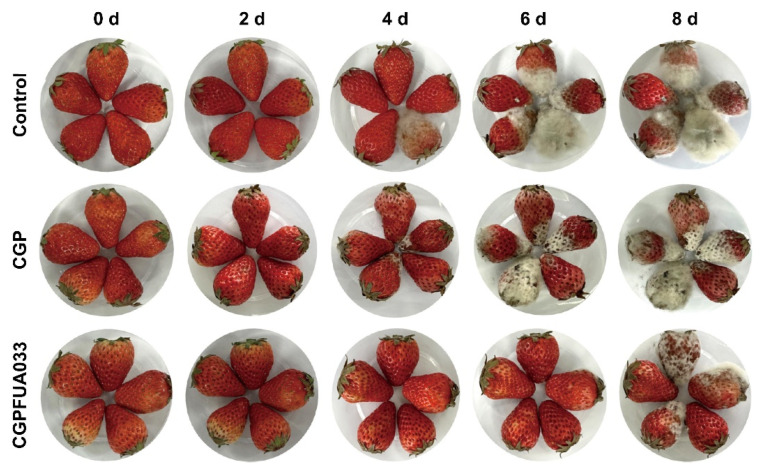
Effects of different treatments on visual quality during strawberry storage.

The presence of surface decay and mold spots on strawberries significantly compromises their edibility and market value, as these defects directly affect visual quality and food safety. By systematically observing changes in appearance, researchers can effectively evaluate the preservation performance of edible coatings [53], with photographic documentation serving as a reliable method of assessment. As shown in Figure 7, the control group of strawberries started showing decay symptoms on the fourth day of storage, characterized by visible mold growth on the fruit surface. By day 6, all samples in the control group had developed distinct mold spots accompanied by noticeable surface wrinkling due to a significant loss of moisture, and complete microbial contamination was observed in all strawberry samples in the control group by day 8. This rapid quality deterioration primarily stems from two characteristics of strawberries: their exceptionally high water content and their delicate, unprotected epidermis, which collectively make them highly vulnerable to mechanical damage during postharvest handling and microbial infection through surface micro-wounds. In contrast, strawberries in the CGP group started showing slight signs of decay on day 4, with the deterioration becoming more pronounced by day 6 and complete decay observed by day 8. The CGPFUA033 group showed optimal preservation outcomes, maintaining fruit plumpness, with only minimal decay symptoms appearing at the end of the storage period. These results indicate that the CGPFUA033 coating not only effectively inhibits the growth of decay-causing microorganisms but also prevents the shrinkage of fruits. This preservation effect can be attributed to the protective film formed by the coating, which maintains the moisture content and suppresses microbial proliferation, thereby preserving the visual freshness of strawberries. Additionally, the extended shelf life and enhanced decay prevention may also result from the continuous inhibitory effect of *L. fermentum* FUA033 metabolites on microbial growth on the surface of strawberries, which significantly delays fruit spoilage and improves storage stability.

## 4. Conclusions

A novel edible film was developed, incorporating the natural antimicrobial and antioxidant probiotic strain *L. fermentum* FUA033, where the bacterial polysaccharides interacted via hydrogen bonds and electrostatic interactions with peptide bonds in the matrix, thereby strengthening the hydrogen bonding network and improving thermal stability of the film. Compared to the control and CGP groups, the CGPFUA033 treatment demonstrated significantly superior preservation performance for strawberries in this study. This group showed substantially reduced strawberry weight loss, pH, TSS, MDA, decay index (DI), and total viable microbial counts, while simultaneously increasing fruit firmness, TA, ascorbic acid retention, and POD activity, ultimately extending the shelf life considerably. These findings highlight the effectiveness of the CGPFUA033 formulation in preserving strawberries and indicate that *L. fermentum* FUA033 can function as both a food freshness-preserving agent and a health-promoting probiotic strain within this specific coating system. The CGPFUA033 film is not a fruit-specific product. Beyond strawberries, its most immediate and significant applications extend to other berries, cherries, grapes, among others. Furthermore, this film holds broad application potential in the fresh-cut fruit industry and is suitable for a variety of products. Tailoring the film thickness or incorporating complementary natural additives could further enhance its fruit-specific efficacy.

## Data Availability

The original contributions presented in this study are included in this article. Further inquiries can be directed to the corresponding author.
